# Calculation of phase envelopes of fluid mixtures through parametric marching

**DOI:** 10.1002/aic.16730

**Published:** 2019

**Authors:** Ulrich K. Deiters, Ian H. Bell

**Affiliations:** 1 Institute of Physical Chemistry, University of Cologne, Köln, Germany; 2 Applied Chemicals and Materials Division, National Institute of Standards and Technology, Boulder, Colorado

**Keywords:** fluid phase equilibria, isochoric thermodynamics, marching algorithm, phase equilibrium calculation

## Abstract

Two-dimensional cross-sections of the phase envelopes of fluid mixtures—in particular isotherms, isobars, and isopleths—are often computed point-by-point by incrementing a so-called marching variable and solving the equilibrium conditions at each step. The marching variable is usually pressure, temperature, or a mole fraction, depending on the application. These isolines, however, can have rather complicated shapes, so that a simple unidirectional “sweep” of the marching variable often gives merely a part of the desired isoline. It is then necessary to restart the sweep with different initial values, or to switch to another marching variable. This, however, makes it difficult to compute complete isolines automatically, without human interference. We propose here a new marching technique through which it is possible to follow isolines of arbitrary shape and thus to compute complete isolines, as long as they are contiguous.

## 1 | INTRODUCTION

Phase equilibria between fluid mixtures—liquid, gas, or supercritical—at elevated pressures play a major role in technology. There are many applications not only in separation processes, but also in power plant and refrigeration technology or in transport processes, where it is essential to know the state of aggregation of a given mixture. Consequently, it is important to develop good models and computational methods for the quantitative description of fluid phase equilibria.

In principle, the phase envelopes of fluid mixtures are objects in $\mathrm{pT}\vec{x}$ space (pressure, temperature, and mole fraction vector). Of course, there exist alternative choices for the primary variables, as will be discussed below, but $\mathrm{pT}\vec{x}$ coordinates are used most frequently. Even for binary mixtures, however, the phase envelopes are three-dimensional objects, and for *N*-component mixtures they are objects in *N* + 1-dimensional space. It is therefore common practice to draw and to discuss two-dimensional cross-sections, in particular isotherms, isobars, and isopleths. For practical purposes, these isolines are computed point-by-point by incrementing an appropriate thermodynamic variable in small steps and each time solving the conditions of phase equilibrium. This thermodynamic variable is called the “marching variable.” Frequently pressure, temperature, or a mole fraction are chosen, depending on the application and the desired isoline.

**FIGURE 1:**
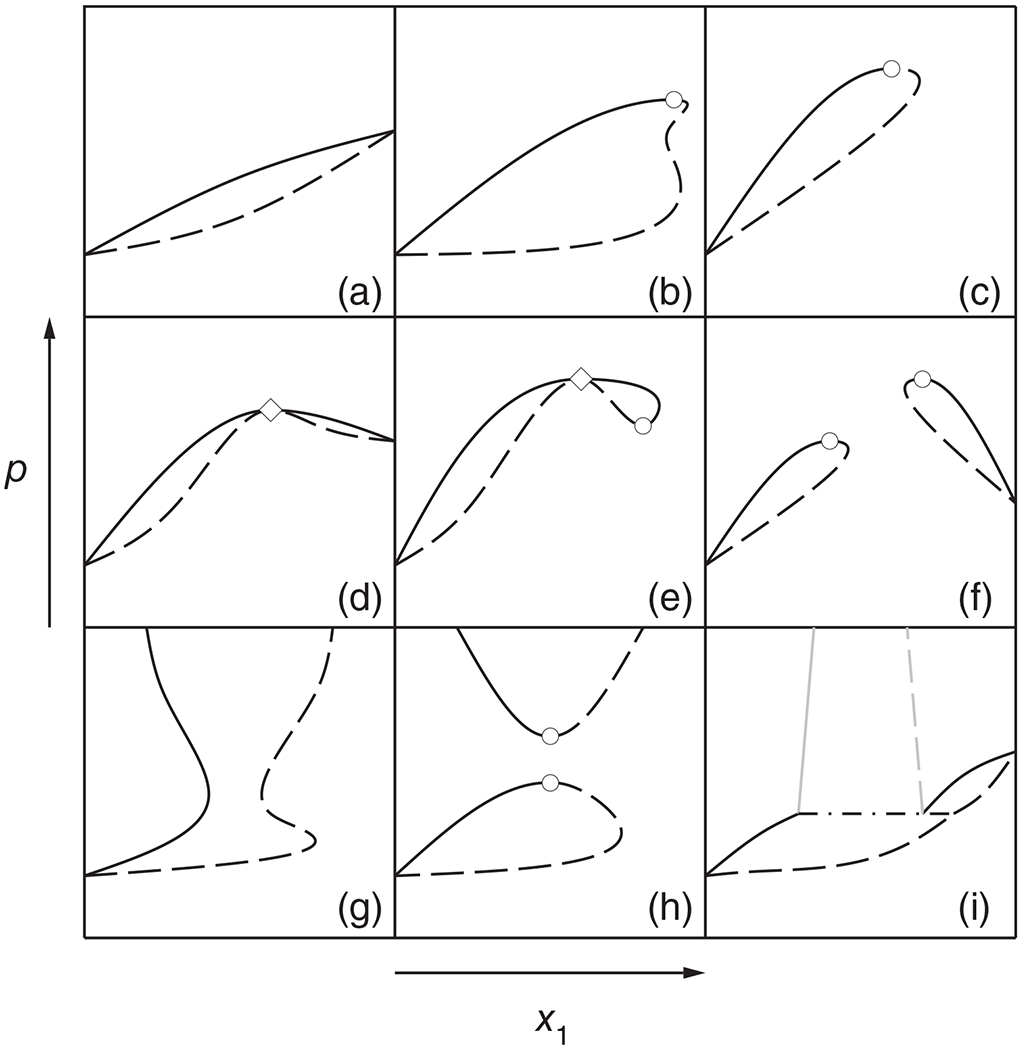
Relevant types of isothermal px phase diagrams of binary fluid mixtures (schematic). —— Bubble point curve, ------ dew point curve, .–.–.– three-phase state, ○ binary critical point, ◇ azeotropic point; gray curves: liquid-liquid equilibrium. See the text for further explanations

These isolines, however, can have rather complicated shapes, particularly if the mixture contains supercritical components. There are many cases where a simple unidirectional “march” does not generate the complete isoline, but merely a part of it. Figure 1 shows a selection of schematic isothermal *px* diagrams for two-component mixtures:

1. plain subcritical case (i.e., both components subcritical)
2. slightly supercritical, with double-retrograde behavior (the “wiggle” of the dew point curve is exaggerated for clarity; usually it is not wider than 1 mol%)
3. plain supercritical case (i.e., Component 1 supercritical, Component 2 subcritical)
4. subcritical case with positive azeotropy
5. system exhibiting positive azeotropy—Component 1 supercritical, Component 2 subcritical
6. system exhibiting positive azeotropy, alternative type—both components subcritical, but above the temperature of the critical azeotropic point
7. gas–gas equilibrium of the second kind, below the temperature minimum of the critical curve
8. gas–gas equilibrium of the second kind, above the temperature minimum of the critical curve
9. superposition of a vapor–liquid and a liquid–liquid equilibrium

The list is far from exhaustive. For sake of brevity, we do not provide a similar list of *Tx* diagram types; the number of the topological varieties is even larger than that of *px* diagrams. Interested readers are referred to reviews and textbooks on this subject.^1–5^

From Figure 1 it is evident that the use of pressure as the marching variable would yield the whole phase diagram for cases (a–c) and (g), but not for cases (d–f), (h), and (i). Conversely, the use of the mole fraction of the liquid phase as the marching variable would give the whole phase diagram for cases (a–d) and (i), but not for (e–h).

It is, of course, important to know whether one can generate the whole isoline with a single sweep of a given marching variable or whether it is necessary to restart the “march” at other conditions, or even to switch to another marching variable, and this question has been considered over the past decades by several authors. For example, Michelsen and his coworkers specified the constant property during a phase envelope calculation by means of an additional equation (see the work of Cismondi and Michelsen^6^ and the literature cited therein), which allowed them to switch the marching variable whenever the slope of the computed curve became too steep. Nikolaides et al^7^ devised a strategy that involves an automatic switching between two different marching variables (the so-called spring-bead method). They demonstrated this method for the calculation of isopleths, but it ought to be possible to extend it to isothermal or isobaric calculations. The strategy requires, however, values of some user control parameters that may not always be known in advance.

Venkatarathnam^8–10^ discussed the use of other thermodynamic properties as marching variables, particularly densities and entropies. It turns out that the liquid molar density or the vapor molar density are often monotonic along phase boundaries—often, but not always. For example, it is well known that many supercritical binary systems (Figure 1c) have density maxima along the liquid branch of the phase envelope, whereas azeotropic systems (d) can be expected to have extrema along the vapor branch. Venkatarathnam showed that monotonic properties, which can be used as marching variables, may even exist for complicated phase diagrams. It is not possible, however, to know which property is the best without some prior knowledge of the mixture under consideration, or without some test calculations.

In this work, we develop a new, different marching strategy, which is able to follow even complicated phase envelopes automatically.

## 2 | THEORY

A good marching algorithm should not only increment the value of the marching variable, but should also provide good initial values for the solver of the phase equilibrium conditions—if these are formulated as a set of algebraic equations. The alternative is a formulation as a set of ordinary differential equations (ODE). Both approaches have their merits, and both can be written most elegantly with the formalism of isochoric thermodynamics.

The formalism has been described elsewhere,^4,11^ but for the readers’ convenience we shall list the most relevant equations below, before proceeding to the description of the new marching method.

### 2.1 | Isochoric thermodynamics

#### 2.1.1 | Definitions and phase equilibrium conditions

The thermodynamic state of a fluid mixture is conventionally characterized by its temperature *T*, its molar volume *V*_m_, and mole fractions *x_i_*;; the associated thermodynamic potential function is the molar Helmholtz energy *A*_m_. In contrast to this, the isochoric thermodynamics formalism uses amounts of substance (“mole numbers”) *n_i_* in a fixed volume *V*, from which the name “isochoric” is derived. Alternatively, one can use molar densities or concentrations as primary variables,

(1) $$\rho_{i}\equiv \frac{n_{i}}{V}=\frac{x_{i}}{V_{m}}=x_{i}\rho ,\;i=1,\ldots ,N.$$

The conversion between the *ρ_i_* and the conventional (*x_i_*, *V*_m_) coordinates is done by

(2) $$\sum_{i=1}^{N}\rho_{i}=\rho =\frac{1}{V_{m}}\;\frac{\rho_{i}}{\rho }=x_{i}.$$

The associated thermodynamic potential is the Helmholtz energy density,

(3) $$\Psi \equiv \frac{A}{V}=\rho A_{m}.$$

The molar Helmholtz energy *A*_m_ consists of a residual and an ideal-gas term. The latter contains chemical contributions as well as contributions stemming from the ideal-gas heat capacities; these, however, are not relevant for phase equilibria and are omitted here.^1^ We can therefore write

(4) $$\Psi =RT\sum_{i=1}^{N}\rho_{i}\ln\left(\frac{\rho_{i}}{\rho^{⊖}}\right)+\Psi^{r}\;\text{with}\;\Psi^{r}\equiv \rho A_{m}^{r},$$

where $A_{m}^{r}$ and $\Psi^{r}$ denote the residual molar Helmholtz energy and the residual Helmholtz energy density, respectively. *ρ*^⊖^ is an arbitrary reference term, for example, *ρ*^⊖^ = 1 mol/cm^3^.

The advantages of using concentrations instead of mole fractions and the molar volume are that (a) the *ρ_i_* have all the same dimension, which makes it easier to define a metric for the $\vec{p}$ space, and (b) that the *ρ_i_* are independent (whereas the *x_i_* must always add up to 1), which allows the usage of vector algebra and considerably simplifies the formulation of equations for multicomponent mixtures.^4,11^

In the isochoric thermodynamics formalism, the chemical potentials are given by

(5) $$\mu_{i}={\left(\frac{\partial \Psi }{\partial \rho_{i}}\right)}_{\rho_{j\neq i},T}=RT\left[1+\ln\left(\frac{\rho_{i}}{\rho }\right)\right]+{\left(\frac{\partial \Psi^{r}}{\partial \rho_{i}}\right)}_{\rho_{j\neq i},T},\;i=1,\ldots ,N$$

and the pressure by

(6) $$p=-\Psi +\sum_{i=1}^{N}\rho_{i}{\left(\frac{\partial \Psi }{\partial \rho_{i}}\right)}_{\rho_{j\neq i},T}=RT\rho -\Psi^{r}+\sum_{i=1}^{N}\rho_{i}{\left(\frac{\partial \Psi^{r}}{\partial \rho_{i}}\right)}_{\rho_{j\neq i},T}.$$

Using vector notation, these two equations can be expressed as

(7) $$\vec{\mu }=\nabla \Psi =RT\ln\;\left(\frac{\vec{\rho }}{\rho }\right)+\nabla \Psi^{r}\;p=-\Psi +\vec{\rho }\cdot \nabla \Psi =RT\rho -\Psi^{r}+\vec{\rho }\cdot \nabla \Psi^{r}.$$

Here the gradient operator (∇) denotes differentiation with respect to all densities, and the “⋅” operator the inner (scalar or “⋅”) product of two vectors.

The conditions of equilibrium between two phases (denoted by *χ* = ′, ″), namely the equality of pressures and the equality of chemical potentials, thus become

(8) $$RT\left[1+\ln\left(\frac{{\vec{\rho }}^{\prime }}{\rho }\right)\right]+\nabla \Psi^{r\prime }=RT\left[1+\left(\frac{{\vec{\rho }}^{\prime \prime }}{\rho }\right)\right]+\nabla \Psi^{r\prime \prime }\;RT\rho \prime -\Psi^{r\prime }+{\vec{\rho }}^{\prime }\cdot \nabla \Psi^{r\prime }=RT\rho \prime \prime -\Psi^{r\prime \prime }+{\vec{\rho }}^{\prime \prime }\cdot \nabla \Psi^{r\prime \prime }.$$

Another frequently needed entity is the Hessian matrix of *Ψ*, which describes the local curvature of the $\Psi (\vec{\rho })$ surface,

(9) $$\begin{array}{l} \Psi \equiv \nabla ⊗\nabla \Psi =\left(\begin{array}{ccc} \Psi_{11} & \ldots & \Psi_{1N}\\ \vdots & \ddots & \vdots \\ \Psi_{N1} & \ldots & \Psi_{NN} \end{array}\right)\\ \begin{array}{cc} \text{with} & \;\Psi_{ij}=\left(\frac{\partial^{2}\Psi }{\partial \rho_{i}\partial \rho_{j}}\right)=\{\begin{array}{ccc} \frac{1}{\rho_{i}}+\left(\frac{\partial^{2}\Psi^{r}}{\partial \rho_{i}^{2}}\right) & \text{if} & i=j\\ \left(\frac{\partial^{2}\Psi^{r}}{\partial \rho_{i}\partial \rho_{j}}\right) & \text{if} & i\neq j \end{array} \end{array}. \end{array}$$

The matrix **Ψ** is symmetric and, for stable states, positive definite.

#### 2.1.2 | Differential equations for isotherms

Of particular importance in the context of this work are the differential equations for phase envelopes.^12–14^ In the formalism of isochoric thermodynamics, the derivatives of the branch of the isothermal phase envelope can be obtained from the following linear matrix equation,

(10) $$\left(\begin{array}{ccc} {\vec{\Psi }}_{1}^{\prime }\cdot {\vec{\rho }}^{\prime \prime } & \ldots & {\vec{\Psi }}_{N}^{\prime }\cdot {\vec{\rho }}^{\prime \prime }\\ {\vec{\Psi }}_{1}^{\prime }\cdot {\vec{\rho }}^{\prime } & \ldots & {\vec{\Psi }}_{N}^{\prime }\cdot {\vec{\rho }}^{\prime }\\ a_{11} & \ldots & a_{1N}\\ \vdots & \ddots & \vdots \end{array}\right){\frac{d{\vec{\rho }}^{\prime }}{d\rho }|}_{T,\sigma }=\left(\begin{array}{c} 1\\ 1\\ 0\\ \vdots \end{array}\right),$$

where ${\vec{\Psi }}_{i}$ stands for the *i*th row vector of **Ψ**. ∣dρ→′∕dp∣T,σ denotes the vector of the derivatives of the concentrations $\rho_{i}^{\prime }$ with respect to pressure at constant temperature and at saturation, that is, while phase equilibrium is maintained. The *a_ij_* are stoichiometric coefficients defining additional conditions: The isothermal phase envelope of an *N*-component mixture has *N* − 1 of freedom. For a ternary mixture it is therefore necessary to impose 1 additional condition to bring the number of degrees of freedom down to 1. Such a condition can be that one mole fraction, for example, $x_{k}^{\prime }$, is kept constant. This can be accomplished by setting

(11) $$a_{1i}=\{\begin{array}{ccc} -x_{k}^{\prime } & \text{if} & i\neq k\\ 1-x_{k}^{\prime } & \text{if} & i=k \end{array}.$$

The *a_ij_* coefficients are needed for ternary or higher mixtures only. The derivation of Equation (11) as well as alternative conditions can be found in the original publication.^12^

Once Equation (10) has been solved for $d{\vec{\rho }}^{\prime }/d\rho ,$ the slopes of the *″* branch can be obtained from another set of linear equations,

(12) $$\Psi^{\prime \prime }{\frac{d{\vec{\rho }}^{\prime \prime }}{dp}|}_{T,\sigma }=\Psi^{\prime }{\frac{d{\vec{\rho }}^{\prime }}{dp}|}_{T,\sigma }.$$

Together, Equations (10) and (12) allow the calculation of all concentration derivatives $d{\vec{\rho }}^{\chi }/dp\left(\chi =\prime ,\prime \prime \right)$ from the given concentrations ${\vec{\rho }}^{\chi }$, so that it is possible to compute the phase envelope by integration.

Equations (10) and (12) can be extended to infinite dilution, so that it is possible to start or end the calculation of an isotherm at a pure-fluid state. The reader is referred to the original publication^12^ for this.

#### 2.1.3 | Differential equations for isobars

The differential equations for isobaric phase envelopes are similar to those for isotherms, except that concentration derivatives with respect to temperature are now calculated, and some derivatives of the chemical potentials and pressure are needed,

(13) $$\left(\begin{array}{ccc} {\vec{\Psi }}_{1}^{\prime }\cdot {\vec{\rho }}^{\prime \prime } & \ldots & {\vec{\Psi }}_{N}^{\prime }\cdot {\vec{\rho }}^{\prime \prime }\\ {\vec{\Psi }}_{1}^{\prime }\cdot {\vec{\rho }}^{\prime } & \ldots & {\vec{\Psi }}_{N}^{\prime }\cdot {\vec{\rho }}^{\prime }\\ a_{11} & \ldots & a_{1N}\\ \vdots & \ddots & \vdots \end{array}\right){\frac{d{\vec{\rho }}^{\prime }}{dT}|}_{p,\sigma }=\left(\begin{array}{c} \left(\Delta \vec{s}\right)\cdot {\vec{\rho }}^{\prime \prime }-\Delta \beta_{\rho }\\ -\Delta \beta_{\rho }\\ 0\\ \vdots \end{array}\right)\;\text{with}\;\Delta \vec{s}\equiv {\vec{s}}^{\prime \prime }-{\vec{s}}^{\prime }={\left(\frac{\partial {\vec{\mu }}^{\prime \prime }}{\partial T}\right)}_{\vec{\rho }}-{\left(\frac{\partial {\vec{\mu }}^{\prime }}{\partial T}\right)}_{\vec{\rho }}\;\Delta \beta_{\rho }\equiv \beta_{\rho }^{\prime \prime }-\beta_{\rho }^{\prime }={\left(\frac{\partial p^{\prime \prime }}{\partial T}\right)}_{\vec{\rho }}-{\left(\frac{\partial p^{\prime }}{\partial T}\right)}_{\vec{\rho }}.$$

and

(14) $$\Psi^{\prime \prime }{\frac{d{\vec{\rho }}^{\prime \prime }}{dT}|}_{p,\sigma }=\Psi^{\prime }{\frac{d{\vec{\rho }}^{\prime }}{dT}|}_{p,\sigma }-\Delta \vec{s}.$$

As in the previous section, the $d{\vec{\rho }}^{\chi }/dT$ denote derivatives along the phase envelope, that is, under saturation, whereas ${\vec{s}}^{\chi }$ and the isochoric tension coefficients $\beta_{\rho }^{\chi }$ are partial derivatives at constant concentrations. Solving Equations (13) and (14)—again, two sets of linear equations—provides values of the $d{\vec{\rho }}^{\chi }/dT,$ which can then be integrated to obtain the phase envelope.

#### 2.1.4 | Differential equations for isopleths

For an isopleth, the composition of one phase is constant, $d{\vec{\rho }}^{\prime }∕dp$. This considerably simplifies the equations for the derivatives,^13^

(15) $${\frac{d{\vec{\rho }}^{\prime }}{dT}|}_{{\vec{x}}^{\prime },\sigma }=\frac{\Delta \vec{s}\cdot {\vec{\rho }}^{\prime \prime }-\Delta \beta_{\rho }}{\left(\Psi \prime \Delta {\vec{\rho }}^{\prime }\right)\cdot {\vec{x}}^{\prime }}{\vec{x}}^{\prime },$$

and

(16) $$\Psi \prime \prime {\frac{d{\vec{\rho }}^{\prime \prime }}{dT}|}_{{\vec{x}}^{\prime },\sigma }=\Psi \prime {\frac{d{\vec{\rho }}^{\prime }}{dT}|}_{{\vec{x}}^{\prime },\sigma }-\Delta \vec{s}.$$

### 2.2 | Normal marching variables

The differential equations presented in the previous sections allow the calculation of density derivatives with respect to pressure or temperature. Therefore the pressure (for the calculation of isotherms) or the temperature (for the calculations of isobars or isopleths) are the primary marching variables. But that does not mean that no other choices are possible. For example, density marching for isothermal calculations can be obtained with the differential equations

(17) $${\frac{d{\vec{\rho }}^{\prime }}{d\rho }|}_{T,\sigma }={\frac{d{\vec{\rho }}^{\prime }}{dp}|}_{T,\sigma }{\left({\frac{d\rho \prime }{dp}|}_{T,\sigma }\right)}^{-1}\;{\frac{d{\vec{\rho }}^{\prime \prime }}{d\rho }|}_{T,\sigma }={\frac{d{\vec{\rho }}^{\prime \prime }}{dp}|}_{T,\sigma }{\left({\frac{d\rho \prime }{dp}|}_{T,\sigma }\right)}^{-1}\;\text{with}\;{\frac{d\rho \prime }{d\rho }|}_{T,\sigma }=\overset{N}{\underset{i=1}{\Sigma }}{\frac{d\rho_{i}^{\prime }}{dp}|}_{T,\sigma }=\vec{1}\cdot {\frac{d{\vec{\rho }}^{\prime }}{dp}|}_{T,\sigma },$$

where the derivatives with respect to pressure are calculated from Equations (10) and (12).

In order to use a mole fraction as the marching variable, it is necessary to consider the definition *ρ_i_* = *x_i_ρ*, which after differentiation yields

(18) $${\frac{d\rho_{i}}{dp}|}_{T,\sigma }=x_{i}{\frac{d\rho }{dp}|}_{T,\sigma }+\rho {\frac{dx_{i}}{dp}|}_{T,\sigma }$$

Rearrangement with *ρ* = ∑_*j*_*ρ_j_* then gives

(19) $${\frac{dx_{i}}{dp}|}_{T,\sigma }={\frac{d\left(\rho_{i}/\rho \right)}{dp}|}_{T,\sigma }=\frac{1}{\rho }{\frac{d\rho_{i}}{dp}|}_{T,\sigma }-\frac{x_{i}}{\rho }{\frac{d\rho }{dp}|}_{T,\sigma }=\frac{1}{\rho }\left[{\frac{d\rho_{i}}{dp}|}_{T,\sigma }-\sum_{j=1}^{N}x_{j}{\frac{d\rho_{j}}{dp}|}_{T,\sigma }\right]$$

and finally the differential equations

(20) $${\frac{d{\vec{\rho }}^{\prime }}{dx_{i}^{\prime }}|}_{T,\sigma }={\frac{d{\vec{\rho }}^{\prime }}{dp}|}_{T,\sigma }{\left({\frac{dx_{i}^{\prime }}{dp}|}_{T,\sigma }\right)}^{-1}\;{\frac{d{\vec{\rho }}^{\prime \prime }}{dx_{i}^{\prime }}|}_{T,\sigma }={\frac{d{\vec{\rho }}^{\prime \prime }}{dp}|}_{T,\sigma }{\left({\frac{dx_{i}^{\prime }}{dp}|}_{T,\sigma }\right)}^{-1}.$$

It should be noted that ${dx_{i}^{\prime }/dp|}_{T,\sigma }$ in this set of equations or d*ρ′*/d*p*|_*T, σ*_ in Equation (17) are not partial derivatives, but derivatives at saturation.

### 2.3 | Parametric marching

The differential equations presented above yield, after integration, the molar concentration vectors of the coexisting phases as functions of pressure (isothermal case) or temperature (isobaric or isoplethic case). In other words, temperature and pressure are the marching variables. But it has already been demonstrated that neither temperature marching nor pressure marching is sufficient for the purposes of this work.

Here we propose an alternative approach that can perform single-sweep scans automatically for phase diagrams of arbitrary shape, as long as the phase boundaries are contiguous. The approach makes use of an auxiliary variable, *t*, which is defined in such a way that it is strictly monotonic along a phase boundary. This can be accomplished by setting the differential of *t* equal to the length of a piece of the phase boundary. For an isothermal phase diagram calculation, a possible choice is

(21) $${\left(dt\right)}^{2}=\sum_{i=1}^{N}{\left(d\rho_{i}\right)}^{2}+{\left(w\;dp\right)}^{2},$$

where *w* is a scaling factor that takes care of the fact that *p* and *ρ_i_* have different units. As there is more than one phase to consider, we set

(22) $$dt=\pm \sqrt{{\left(w\;dp\right)}^{2}+{\left(d{\vec{\rho }}^{\prime }\right)}^{2}+{\left(d{\vec{\rho }}^{\prime \prime }\right)}^{2}}.$$

Here the Exponent 2, if applied to a vector, denotes the scalar product with itself or, in other words, the square of the Euclidean norm,

(23) $${\left(d{\vec{\rho }}^{\chi }\right)}^{2}=\left(d{\vec{\rho }}^{\chi }\right)\cdot \left(d{\vec{\rho }}^{\chi }\right)=\sum_{i=1}^{N}{\left(d\rho_{i}^{\chi }\right)}^{2},\chi =\prime ,\prime \prime .$$

For the purpose of this work, the value of *w* is arbitrary and can be set to zero. Division by d*p* then yields

(24) $$\frac{dt}{dp}=\pm \sqrt{{\left(\frac{d{\vec{\rho }}^{\prime }}{dp}\right)}^{2}+{\left(\frac{d{\vec{\rho }}^{\prime \prime }}{dp}\right)}^{2}},$$

and the differential equations of an isotherm therefore become

(25) $${\frac{d{\vec{\rho }}^{\prime }}{dt}|}_{T,\sigma }={\frac{d{\vec{\rho }}^{\prime }}{dp}|}_{T,\sigma }{\frac{dp}{dt}|}_{T,\sigma }\;{\frac{d{\vec{\rho }}^{\prime \prime }}{dt}|}_{T,\sigma }={\frac{d{\vec{\rho }}^{\prime \prime }}{dp}|}_{T,\sigma }{\frac{dp}{dt}|}_{T,\sigma }\;{\frac{dp}{dt}|}_{T,\sigma }=\pm {\left({\left({\frac{d{\vec{\rho }}^{\prime }}{dp}|}_{T,\sigma }\right)}^{2}+{\left({\frac{d{\vec{\rho }}^{\prime \prime }}{dp}|}_{T,\sigma }\right)}^{2}\right)}^{-\frac{1}{2}}.$$

The $d{\vec{\rho }}^{\chi }/dp$ are of course obtained from the differential equations, in this case Equations (10) and (12).

There remains however the problem of determining the proper sign of d*p*/d*t*. This can be accomplished in the following way:

1. The integration starts at a given initial state to which a *t* value of zero is assigned that is
  - *t*_0_ = 0,
  - ${\vec{\rho }}^{\prime }\left(t_{0}\right)$ and ${\vec{\rho }}^{\prime \prime }\left(t_{0}\right)$ are known, so that $d{\vec{\rho }}^{\prime }/dp$ and $d{\vec{\rho }}^{\prime \prime }/dp$ can be computed,
  - and the sign of d*p*/d*t* can be chosen freely depending on whether one wishes to follow the phase boundary to lower or to higher pressures.
2. In order to integrate the set of differential equations from *t*_*k*−1_ to *t_k_*, *k* > 0, it is necessary to evaluate Equation (25) at several intermediate locations. If *t_k_* − *t*_*k*−1_ is sufficiently small, one may assume that the derivatives $d{\vec{\rho }}^{\chi }∕\mathrm{dp}\;(\chi {=}^{\prime }{,}^{\prime \prime })$ at these locations are similar; in particular one may assume that these vectors have similar directions and their scalar product is therefore positive. A simple recipe for determining the sign of d*p*/d*t* is then to
  - evaluate Equation (25), tentatively with the positive sign,
  - evaluate the criterion
    (26) $$c={\frac{d{\vec{\rho }}^{\prime }\left(t_{k-1}\right)}{dt}|}_{p,\sigma }\cdot {\frac{d{\vec{\rho }}^{\prime }\left(t\right)}{dt}|}_{p,\sigma }>0$$
  - and if *c* is negative use the negative sign in Equation (25).

The equations for parametric marching presented in this section are for isotherms. But merely by swapping *p* and *T*, the method can be extended to isobars or isopleths.

### 2.4 | Programming considerations

Equation (25) can be integrated by means of standard integration methods for ODE using adaptive step sizing. For the work presented here, the method of Cash and Karp^15^ was used.

In order to prevent the accumulation of round-off errors—a typical problem of ODE integrators—it is advisable to follow each integration step by one or more steps of a Newton–Raphson or Marquardt–Levenberg solver (applied to the algebraic phase equilibrium condition, Equation (8)).^14^ Whether one considers the parametric-marching algorithm an ODE-based approach that uses an algebraic solver for the improvement of its results, or an algebraic-equation based approach that uses ODEs to generate initial values, is a matter of taste. The important fact is that the combination of ODEs and algebraic equations makes the new approach possible.

It is important to remember that the algebraic criterion for phase equilibrium, Equation (8), as well as all differential equations, become undefined at a critical point. Moreover, they become unreliable in the immediate vicinity of a critical point due to unfavorable cancelations of terms. Most likely, the ODE integrator will pass through a critical point with a loss of precision, and then the algebraic solver will restore the precision again. If this fails, it is usually sufficient to “jump over” the critical point by extrapolating the last two good concentration vectors, for example, by using ${\vec{\rho }}^{\chi }=2{\vec{\rho }}_{k}^{\chi }-{\vec{\rho }}_{k-1}^{\chi }$.

Neither azeotropic points nor maxcondentherm and maxcondenbar points are special states from the viewpoint of thermodynamic conditions. At such points, the coexisting phases usually have different densities, and the phase equilibrium conditions as well as the differential equations can be evaluated without problems.

By replacing the last sub-equation of Equation (25) by

(27) $${\frac{dp}{dt}|}_{T,\sigma }=1,$$

the marching algorithm can be forced to fall back to standard, unidirectional marching.

Algorithm 1 briefly summarizes the computation steps for parametric marching. We emphasize that the method can be applied to any equation of state or mixing rule. The equation of state is only used “in the forward direction”, that is, for the calculation of the pressure for a given density vector. The reverse calculation, namely the calculation of the density for a given pressure, is never needed, which is advantageous when noncubic equations of state are used.

**Algorithm 1:** Outline of the algorithm for parametric marching (isothermal case).

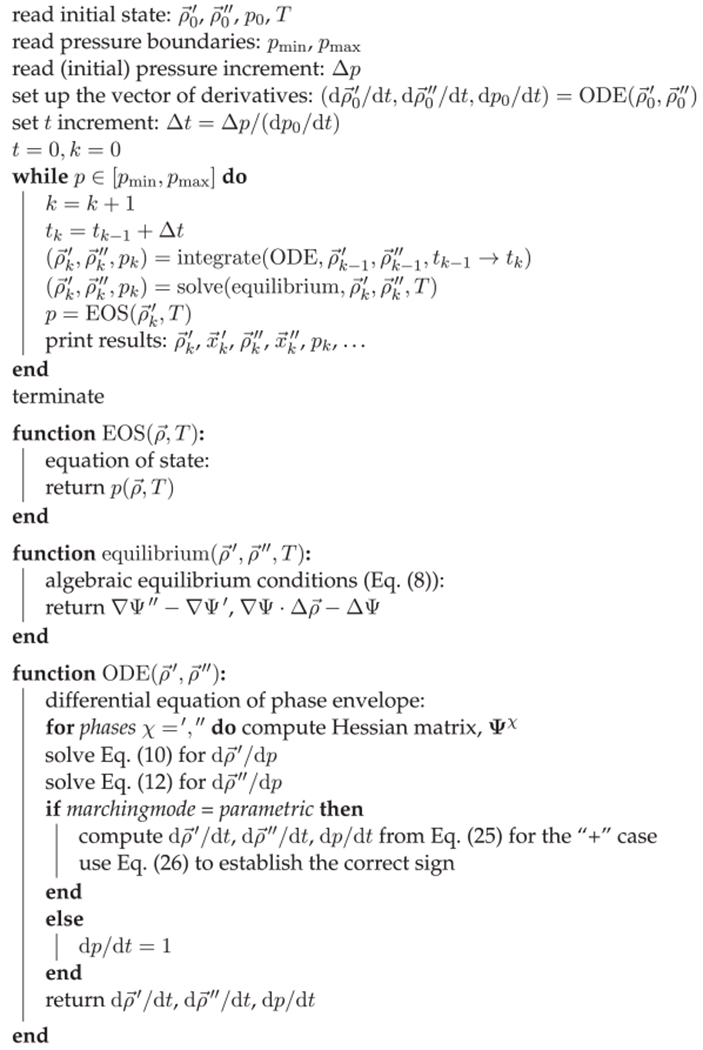

## 3 | APPLICATION

### 3.1 | The (methane + propane) system

**FIGURE 2:**
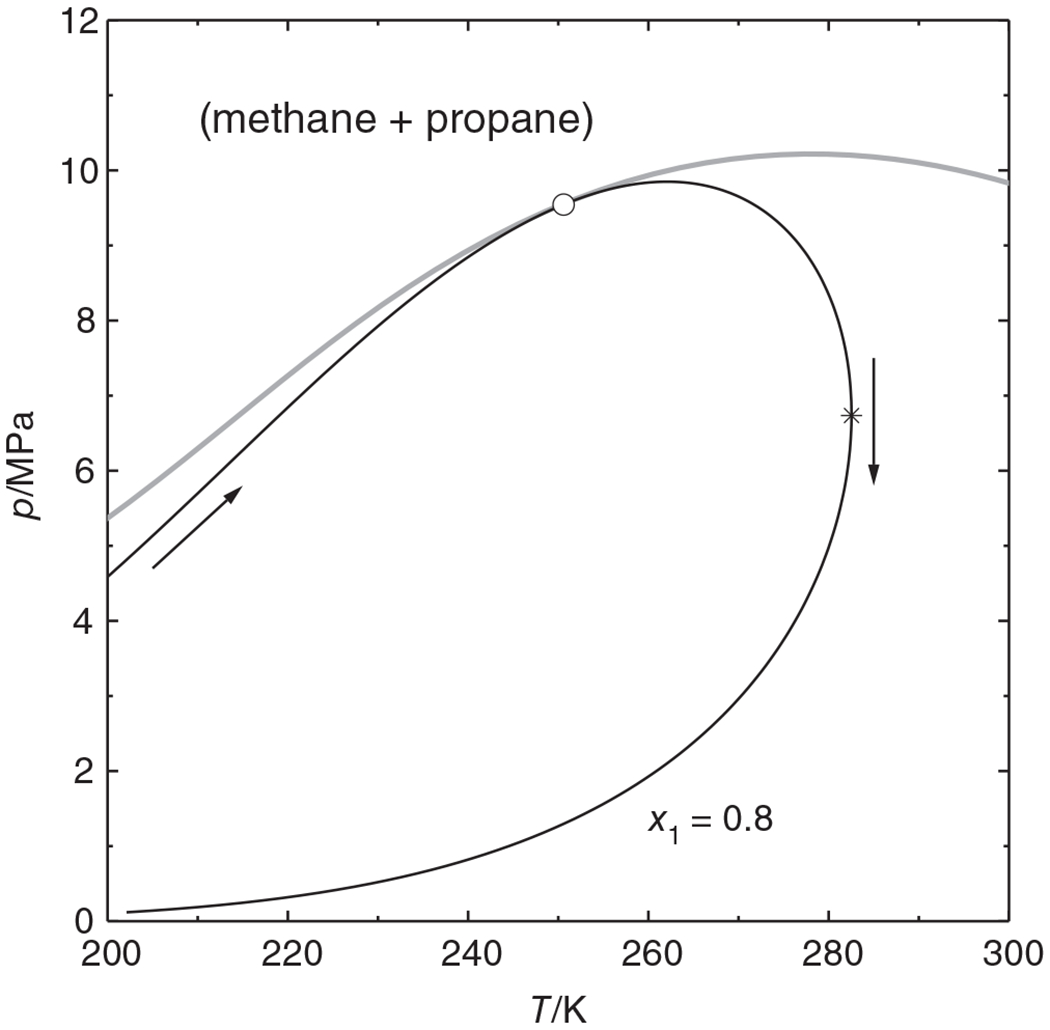
Isopleth *x*_1_ = 0.80 of the (methane + propane) system, calculated with the Peng–Robinson equation of state. —— Isopleth; ○ mixture critical point; * maxcondentherm point; gray curve: (projection of) critical curve. The arrows indicate the marching direction

We wish to demonstrate the method of parametric-marching method through the calculation of an isopleth of the binary (methane + propane) system. The thermodynamic model in this case is the Peng–Robinson equation with the one-fluid mixing rules proposed by its authors^16^ and parameters fitted to experimental data by Reamer et al.^17^ The values of the parameters are shown in Table 1. In the temperature range considered here (above 200 K), methane is supercritical. The *x*_1_ = 0.8 isopleth passes through a critical point at about 251 K and then through a maxcondentherm point at 282 K before turning back to low temperatures (cf. Figure 2). Table 2 shows the evolution of the concentration derivatives in the vicinity of the maxcondentherm point, where, just at this point, the criterion in Equation (26) becomes negative, and this causes *dT/dt* to change sign and thus the isopleth to turn back.

**FIGURE 3:**
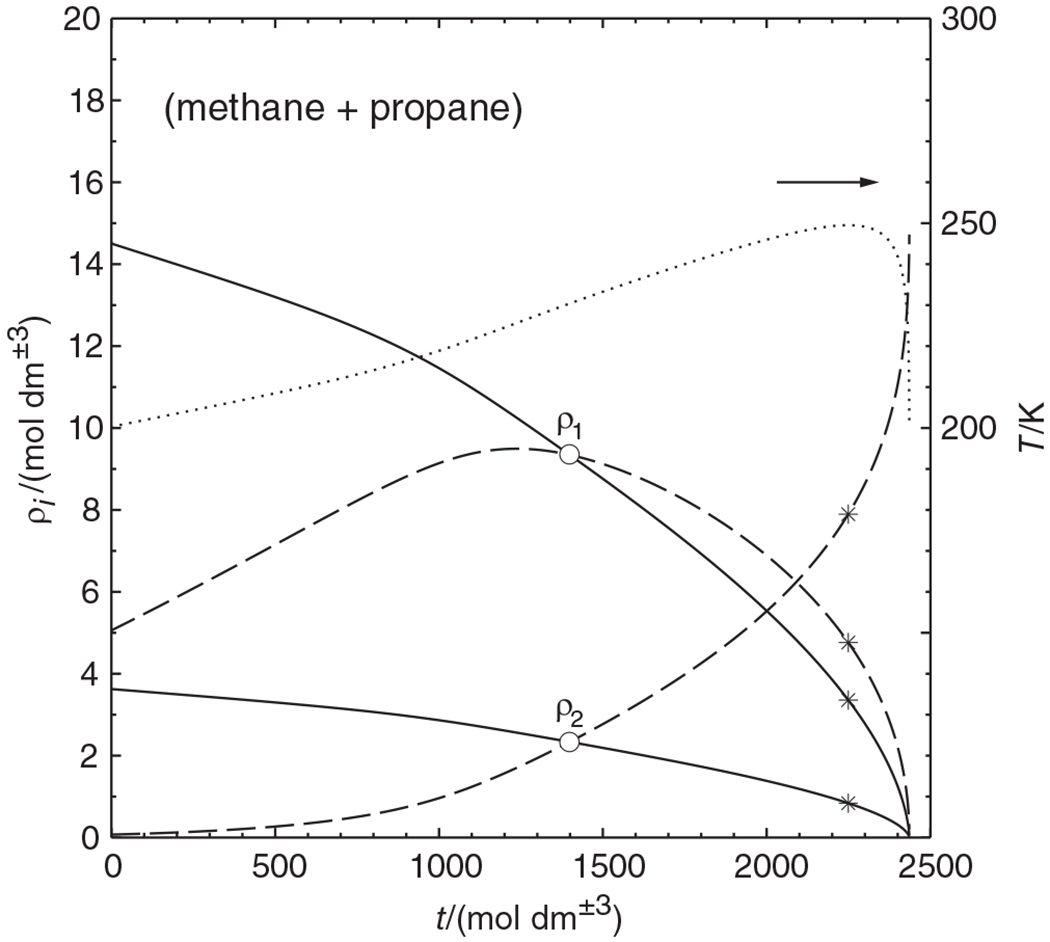
Equilibrium concentrations *ρ_i_* and temperature of the (methane + propane) system along the *x*_1_ = 0.80 isopleth shown in Figure 2 as function of the parametric marching variable. —— $\rho_{i}^{\prime }$ (concentrations of the phase with the fixed composition); ------ $\rho_{i}^{\prime \prime }$ (concentrations of the coexisting phase), .–.–.– temperature; ○ critical point, * maxcondentherm point

Figure 3 shows the concentrations of the coexisting phases as a function of the parametric marching variable *t*. The calculation starts at *t* = 0 and proceeds to the right. The concentrations are smooth and unique functions of *t* and pass through the maxcondentherm point without any problems; the “loop” appears only when the results are plotted versus temperature, as in Figure 2.

The parametric marching proposed here makes use of the local continuity of molar densities. In a way, it is therefore similar to Venkatarathnam’s density marching method.^8,10^ It is, however, more flexible (unless switching strategies are added to the density marching method).

**FIGURE 4:**
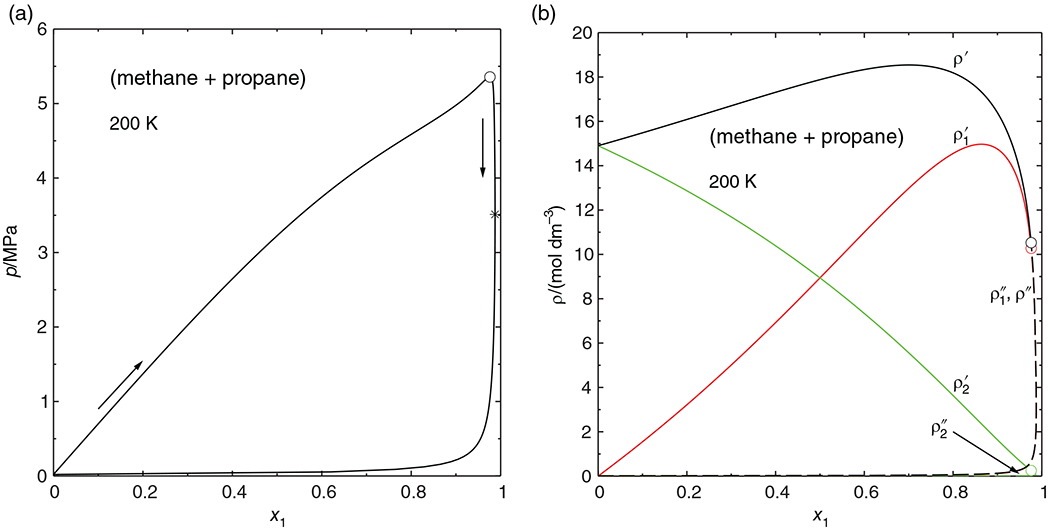
Isothermal phase diagram of the (methane + propane) system at 200 K, calculated with the Peng–Robinson equation of state. ○ mixture critical point; * maxcondentherm point. (a) Pressure versus mole fractions $x_{1}^{\prime }$, $x_{1}^{\prime \prime }$ of methane. (b) Molar concentrations *ρ*_1_, *ρ*_2_ and total density of liquid (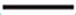) and vapor (------) [Color figure can be viewed at wileyonlinelibrary.com]

As shown in the previous section, parametric marching is not restricted to isopleths. Figure 4a shows an isothermal phase diagram of the (methane + propane) system at 200 K, calculated as before with the Peng-Robinson equation of state. For this system the pressure as well as the methane mole fraction of the liquid phase are good marching variables, that is, by monotonically increasing one of these variables, the complete phase envelope can potentially be obtained. A normal pressure march must naturally terminate short of the critical point, which leaves a gap in the phase envelope (a phenomenon seen in many publications); a normal march with liquid mole fraction must evidently end just short of the maxcondentherm state, which usually causes another gap in the phase envelope. With parametric marching, the phase envelope can be followed through the critical point and the maxcondentherm point.

Figure 4b reveals that the liquid density has a maximum along the phase envelope. This is a rather common phenomenon with near- or supercritical phase diagrams. It means, however, that Venkatarathnam’s method (density marching with the liquid density) would not yield the complete envelope here, nor would $\rho_{1}^{\prime }$ be a good marching variable. Instead, $\rho_{2}^{\prime }$ would be a good marching variable (decreasing monotonically along the phase envelope). This, however, is only known *after* performing several phase envelope calculations, whereas parametric marching generates the whole phase envelope in a single sweep.

### 3.2 | The system (carbon dioxide + ethane)

**FIGURE 5:**
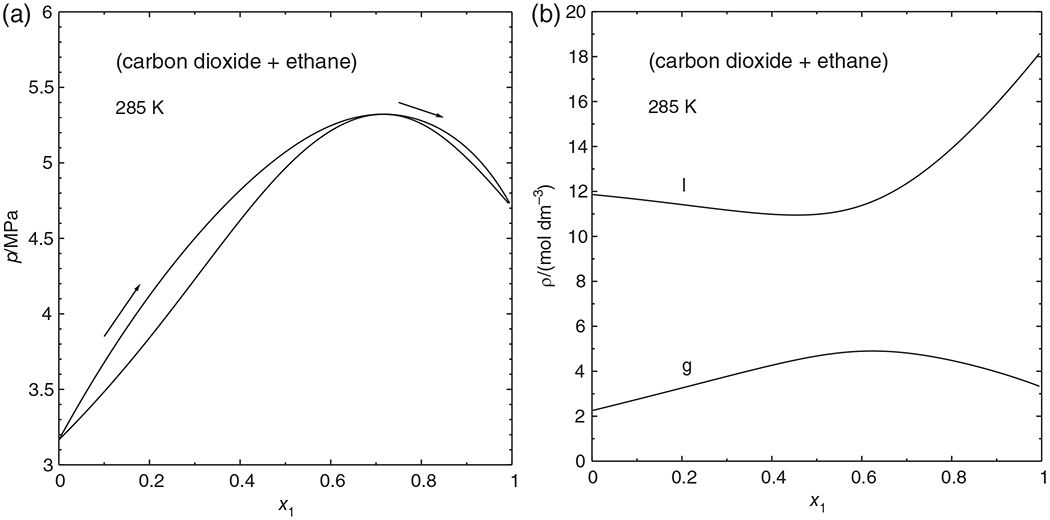
Isothermal phase diagram of (carbon dioxide + ethane) at 285 K, calculated with the Peng–Robinson equation of state. (a) Pressure versus mole fraction of carbon dioxide. (b) Liquid (I) and vapor (g) density versus $x_{1}^{\chi }$

Figure 5a shows an isothermal phase diagram for the azeotropic system (carbon dioxide + ethane) at 285 K, computed with the Peng–Robinson equation of state,^16^ with parameters fitted to experimental data of Ohgaki and Katayama.^18^ Parametric marching can generate the phase envelope in a single sweep. Simple density marching could not accomplish this, for neither the liquid density nor the vapor density is monotonic along the phase envelope, as can be seen in Figure 5b.

**FIGURE 6:**
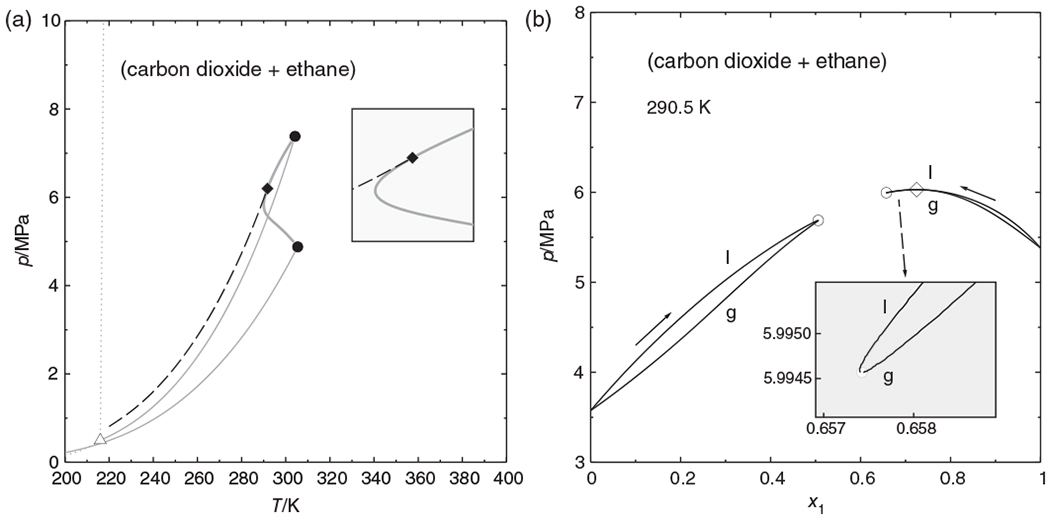
Phase behavior of the (carbon dioxide + ethane) system, calculated with the Peng–Robinson equation of state. (a) *p*, *T* phase diagram. ------ azeotropic curve, ● pure-fluid critical point, ◆ critical azeotropic point, Δ triple point (CO_2_); 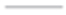 grey curves: 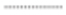 vapor pressure curve, 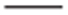 critical curve, ------ melting or sublimation pressure curve (CO_2_). (b) Isothermal phase diagram at 290.5 K. ◇ azeotropic point, ○ mixture critical points

The critical temperatures of carbon dioxide and ethane are almost equal (304.21 and 305.4 K, respectively), and the critical curve of the (ethane + carbon dioxide) mixture passes through a temperature minimum, as can be seen in Figure 6a. With the set of model parameters used in this work, the minimum lies at 290.06 K. The critical azeotropic point—the osculating point of the azeotropic and the critical curve—lies not at this minimum, but at a slightly higher temperature, namely at 291.74 K. Between these temperatures, the *px* phase diagram (Figure 6b) has a rather complicated shape, which may be regarded as a combination of Figure 1e,f: There are two separate phase envelopes. The one starting on the ethane side has the typical shape of a supercritical isotherm—although both components are subcritical—with a critical point at its *maximum*. The phase envelope starting on the carbon dioxide side passes through a shallow pressure maximum, an azeotropic point. After this point, the bubble point curve runs to lower pressures and mole fractions, as shown in the inset of Figure 6b, turns to higher mole fractions, and finally meets the dew point curve at a critical point that is a pressure *minimum*. Evidently, a phase envelope with such a shape cannot be obtained by either pressure or mole fraction marching alone. With parametric marching, however, the whole phase envelope could be computed in a single sweep.

We wish to emphasize that a simple marching strategy with a too coarse step width might have “jumped” from one envelope to the other, thus missing the critical points and predicting a phase split where there is none. In contrast to this, the parametric marching algorithm reduces its step width and follows the equilibrium curves even when they change directions.

### 3.3 | The system (tetrafluoromethane + butane)

**FIGURE 7:**
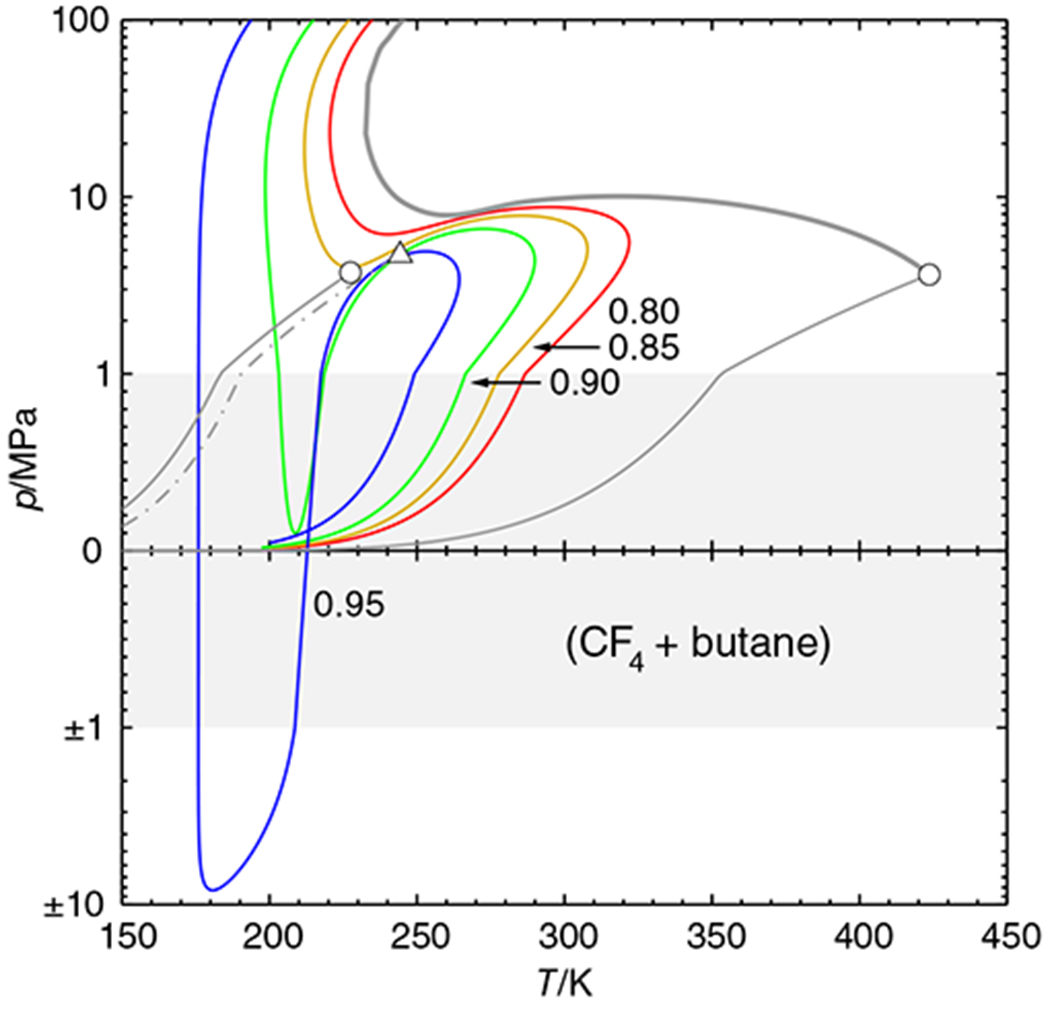
Phase diagram (with a “symmetric logarithmic” pressure axis) of the system (tetrafluoromethane + butane), computed with the Peng–Robinson equation of state. Color lines: isopleth (parameter: mole fraction *x*_1_), ● pure-component critical point, Δ upper critical endpoint; 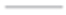 grey curves: —— vapor pressure curve, 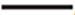 critical curve, .—.—.— three-phase curve [Color figure can be viewed at wileyonlinelibrary.com]

According to experimental evidence by Jeschke and Schneider,^19^ the system (tetrafluoromethane + butane) has a phase diagram with an interrupted critical curve (tetrafluoromethane: CF_4_, R-14). Figure 7 shows that the branch of the critical curve that originates at the critical point of butane first runs to lower temperatures, but then turns to higher temperatures again; the turning point is at about 230 K and 35 MPa. The diagram has a “symmetric logarithmic” pressure axis (linear between −1 and + 1 MPa, otherwise logarithmic) in order to better accommodate the wide pressure range covered by the calculations, which were made with the Peng-Robinson equation of state.^16^ Several isopleths have been plotted in the diagram. The isopleths for *x*_1_ = 0.80–0.90 have both temperature and pressure minima and maxima, which means that they change their direction with respect to temperature twice. The *x*_1_ = 0.95 isopleth even forms a loop and passes through a metastable region of negative pressures. The new marching method could obtain all these curves in a single sweep.

In other test calculations for binary and ternary mixtures, the algorithm was found to proceed smoothly through azeotropic points (including cases of double azeotropy), critical points, and maxcondentherm and maxcondenbar points (including cases of double retrogade behavior) without manual assistance.

**FIGURE 8:**
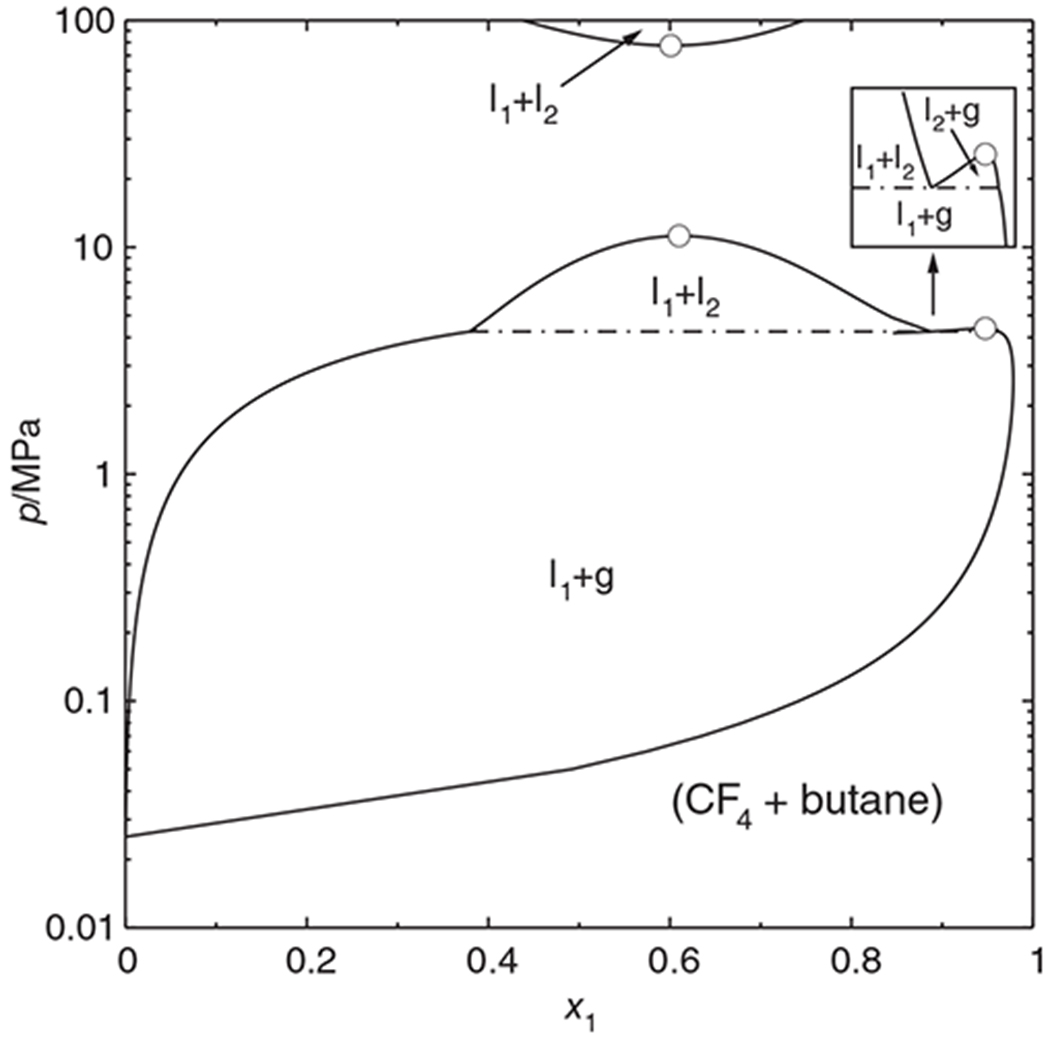
Isothermal phase diagram of (tetrafluoromethane + butane) at 240 K, calculated with the Peng–Robinson equation of state. 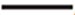 Phase envelope, .—.—.— three-phase state, ○ mixture critical point

This does not mean, however, that the parametric marching method will always and automatically obtain complete fluid phase diagrams. For example, the isothermal *px* diagram of (carbon dioxide + ethane) at 302 K conforms to the phase diagram type in Figure 1f. One can conclude from the fact that the left phase envelope does not extend to *x*_1_ = 1 although both components are subcritical that there must be another phase envelope—but it cannot be found by parametric marching; instead one has to restart the program at the other side or trace the critical curve for a while. Another problem case would be the phase diagram shown in Figure 8. The temperature of this diagram lies between the critical temperature of the volatile compound and the upper critical endpoint (cf. Figure 7), so that there are vapor–liquid as well as liquid–liquid coexistence regions. A parametric march started at the vapor pressure of butane would stop at the three-phase state.^2^ This behavior is necessary for physical reasons, for there is no valid vapor–liquid phase envelope beyond the three-phase state, but this means that a part of the phase diagram cannot be obtained by simply “marching on.” Instead, it is necessary to find the third equilibrium phase—the construction of an eigenvector path^11^ starting at one of the equilibrium states usually helps to locate it—and then to restart the phase envelope calculation manually.

But even with such additional measures it would be easy to overlook the high-pressure phase separation above 70 MPa. This points to a pitfall of the parametric marching method of which its users should be aware: The method uses *local* properties of the Ψ surface to find the desired phase envelope. The method is not “aware” of phase separations occurring elsewhere. It is therefore advisable to first establish the phase diagram class by computing the critical curve(s) of the mixture under consideration; there are algorithms that can locate all critical lines of a mixture^4,20^ and which would, in particular, reveal the existence of three critical points in the phase diagram, as shown in Figure 8. In a second step, one can then compute the phase envelopes with the parametric marching method.

This corroborates the observations of Cismondi and Michelsen,^6^ who considered strategies for computing complete phase diagrams and particularly gave thought to phase diagrams with liquid–liquid–vapor three-phase states.

### 3.4 | The system (helium + xenon)

**FIGURE 9:**
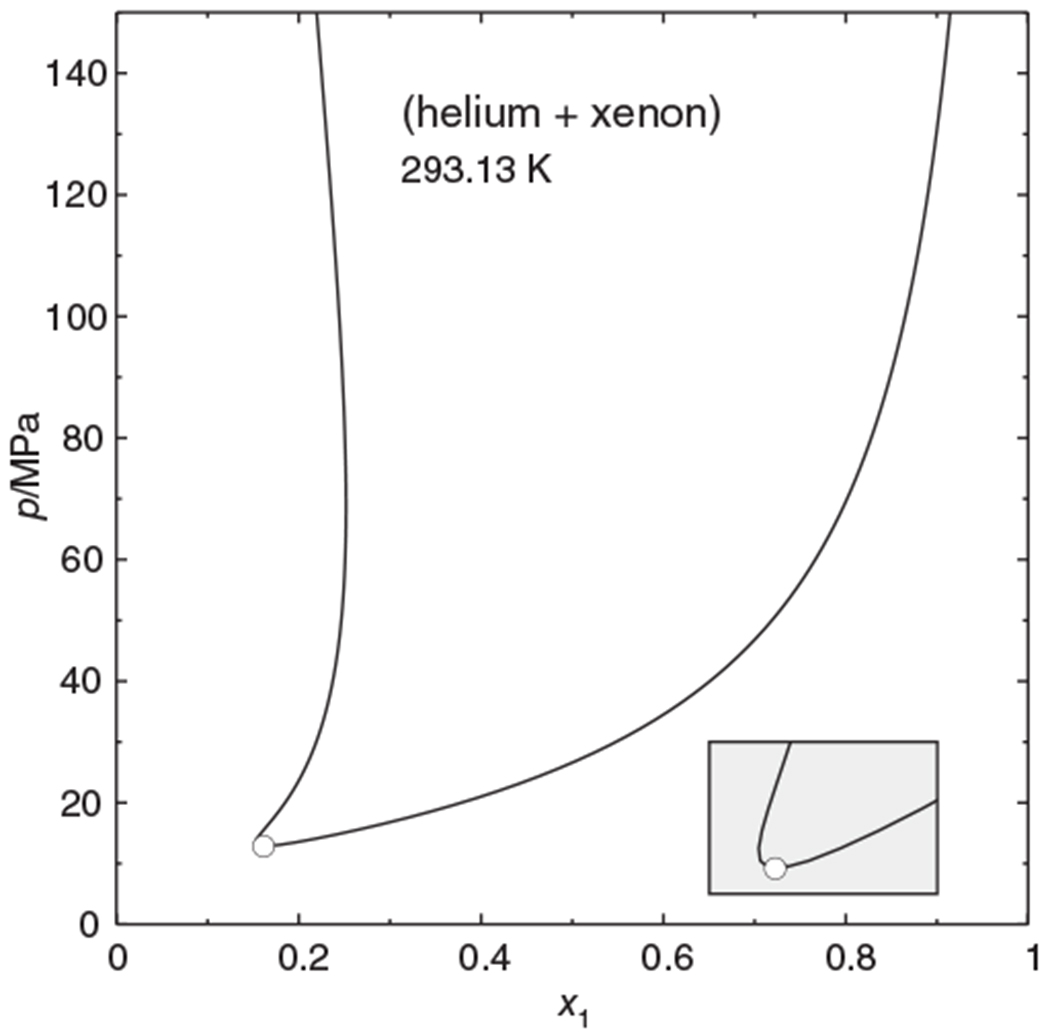
Isothermal phase diagram of the (helium + xenon) system at 293.13 K, calculated with the Peng–Robinson equation of state. — Phase envelope, ○ mixture critical point. Inset: enlargement of the critical region

A famous case of a phase separation region not connected to the pure-component vapor pressure curves is the system (helium + xenon), which exhibits a so-called gas–gas equilibrium of the first kind. The isothermal phase envelope shown in Figure 9 was calculated with the Peng–Robinson equation of state with parameters fitted to the experimental data^3^ of de Swaan Arons and Diepen.^21^ The temperature of the isotherm is 293.13 K, which is above the critical temperature of both components. The two-phase region is topologically related to the high-pressure two-phase region in Figure 8, but now the phase boundary of the xenon-rich phase is S-shaped. The parametric-marching algorithm, starting at high pressures and using $x_{1}^{\prime }$ as marching variable, could follow the double bend without difficulties. In this case pressure marching would also have been adequate, of course. But one can know this only *after* having computed the phase diagram.

This argument can be generalized:

- Of course each phase envelope can be assembled from pieces obtained by conventional pressure, temperature, or mole fraction marching. This can be tedious work, however, if the numbers of these pieces and the locations of their start and endpoints have yet to be determined.
- Even if the locations of the critical points in a *px* or *Tx* phase diagram are known from analyzing the pattern of critical curves and establishing the phase diagram class, this knowledge is not always sufficient to predict the existence—even less the locations—of the “turning points” of isobars, isotherms, or isopleths.
- While the computation of complete phase envelopes by conventional marching is not always easy for binary mixtures, it can get very complicated for multicomponent mixtures.

Parametric marching, on the other side, is always applicable.

One might wonder whether it is really necessary to use the differential equations instead of a simple extrapolation scheme based, for instance, on polynomials. Our experience is, however, that the differential equations provide very good initial values for the algebraic solver—much better than polynomial extrapolation schemes. Such schemes were frequently found to “get derailed” around maxcondentherm points, whereas the differential equations can follow isolines even in difficult cases because of their built-in thermodynamics.

## 4 | CONCLUSION

Parametric marching uses not a thermodynamic variable, but an auxiliary control variable for running along phase envelopes. This auxiliary variable is computed from the arc length of the computed phase envelope and therefore is strictly monotonic under all circumstances—which cannot be said for thermodynamic properties. This auxiliary variable is based on the concentrations (molar densities) of the coexisting phases.

The parametric marching method makes use of differential equations for phase envelopes and then uses an algebraic solver to eliminate round-off errors. Both parts of the calculation can be expressed with the formalism of isochoric thermodynamics. This facilitates the implementation in computer programs, particularly if a programming language is used that supports vector and matrix operations.

The parametric marching method does not use any control parameters whose determination would require prior knowledge of the phase diagram or necessitate test calculations.

It is thus possible to calculate all contiguous phase envelopes from Figure 1 in a single sweep of the marching variable. Phase diagrams containing noncontiguous phase boundaries still need special precautions. When dealing with phase diagrams of unknown type, it is advisable to calculate the critical curves first, because this will already provide an overview over the possible shapes of isotherms, isobars, and isopleths.

That phase equilibrium algorithms based on integrating differential equations are more reliable and often faster than conventional methods has already been established in previous publications.^12–14^ Using parametric marching instead of marching with a thermodynamic property will probably not reduce the CPU time. It will reduce, however, the human effort needed to reconsider the choice of marching variables and to restart computer runs whenever a phase equilibrium curve changes its direction.

## NOTATION

**Table:** 

*A*	Helmholtz energy
*a_ij_*	stoichiometric coefficient, condition #*i* for *ρ_j_*, Equation (11)
*c*	switching criterion, Equation (26)
*k*_12_	binary interaction parameter of the Peng–Robinson equation of state
*N*	number of components of a mixture
*n_i_*	amount of substance of component *i*
*p*	pressure
*s_i_*	temperature derivative of the chemical potential, $s_{i}={\left(\partial \mu_{i}/\partial T\right)}_{\vec{\rho }}$
$\vec{s}$	vector of *s_i_*, $\vec{s}=\left(s_{1},\ldots ,s_{N}\right)$
*T*	temperature
*t*	parametric marching variable
*V*	volume
*w*	scaling factor (here *w* = 0)
*x_i_*	mole fraction of component *i*
$\vec{x}$	vector of mole fractions, $\vec{x}=\left(x_{1},\ldots x_{N}\right)$
*β_ρ_*	isochoric tension coefficient, $\beta_{\rho }={\left(\partial p/\partial T\right)}_{\vec{\rho }}$
*μ_i_*	chemical potential of component *i*
$\vec{\mu }$	vector of chemical potentials
*ρ*	molar density, $\rho \equiv V_{m}^{-1}$
*ρ_i_*	density (concentration) of component *i*
$\vec{\rho }$	vector of concentrations
**Ψ**	Helmholtz energy density, **Ψ** ≡ *A/V*
*ω*	acentric factor (parameter of the Peng–Robinson equation of state)

## SUBSCRIPTS

**Table:** 

c	critical property
m	molar property
*σ*	at saturation

## SUPERSCRIPTS

**Table:** 

*r*	residual property
*χ*	generic phase label
*′,″*	phase labels

## MATHEMATICAL NOTATION

**Table:** 

${\left(\vec{\text{v}}\right)}^{2}$	squared Euclidean norm of vector $\vec{\text{v}},{\left(\vec{\text{v}}\right)}^{2}=\vec{\text{v}}\cdot \vec{\text{v}}=\sum_{i}{\text{v}}_{i}^{2}$
${\frac{dY}{dX}|}_{Z,\sigma }$	derivative of *Y* w.r.t. *X* at constant *Z* at saturation (i.e., along the phase boundary)

## Figures and Tables

**TABLE 1:** Parameters of the Peng–Robinson equation of state used in this work (critical pressure and temperature, acentric factor, binary interaction parameter)

Substance	*p*_c_/MPa	*T*_c_/K	*ω*	*k*_12_
methane	4.595	190.555	0.0	
ethane	4.88	305.4	0.099	
propane	4.248	369.825	0.15308	
butane	3.6348	423.675	0.19290	
CF_4_	3.7389	227.5	0.18884	
CO_2_	7.3825	304.21	0.228	
helium^a^	0.29738	5.47956	0.0	
xenon	5.8405	289.765	0.0	
methane + propane				0.042823
CO_2_ + ethane				0.136845
CF_4_ + butane				0.21901
helium + xenon^b^				0.99036

a Effective critical data fitted to *pVT* data in the range 30–100 K.

b An additional interaction parameter for the covolume was used, *I*_12_ = −0.08.

**TABLE 2:** Evolution of the density derivatives $d\rho_{i}^{\chi }∕\mathrm{dT}$ the switch criterion, Equation (26), and the derivative of temperature with respect to the parametric variable *t*, in the vicinity of the maxcondentherm point, calculated with the Peng–Robinson equation of state (density units d.u. = mol/dm^3^)

T	$d\rho_{1}^{\prime }∕\mathrm{dT}$	$d\rho_{2}^{\prime }∕\mathrm{dT}$	$d\rho_{1}^{\prime \prime }∕\mathrm{dT}$	$d\rho_{2}^{\prime \prime }∕\mathrm{dT}$	10^−6^c	*dT/dt*
K	d.u./K	d.u./K	d.u./K	d.u./K	(d.u./K)^2^	K/d.u.
282.51	−2.34899	−0.58725	−2.50076	2.84777	0.292	222.353

282.53	−7.47457	−1.86864	−8.02569	9.17418	0.288	69.348

282.53	5.89304	1.47326	6.38176	−7.32422	−0.283	−87.281

282.50	2.06003	0.51501	2.25000	−2.59312	−0.279	−247.721

## Footnotes

1 They must be included for studies of non-isothermal processes or chemical equilibria.

2 More accurately: slightly after the three-phase state, for there is a metastable region between the three-phase state and the mathematical terminus.

3 Helium cannot be described well with the Peng–Robinson equation of state. Parameters fitted to experimental data usually show a strong temperature dependence; an interpretation of their values should not be attempted.
